# Supplementary material for: Optimizing bike-sharing station locations: A machine learning and artificial neural networks approach using geospatial and demographic data
Source: PLoS One. 2026 May 19;21(5):e0349339. doi: 10.1371/journal.pone.0349339 (PMC13186375; doi:10.1371/journal.pone.0349339)
Supplement: S1 Table — (DOCX) [file pone.0349339.s001.docx]

| **Factor** | **Data origin** | | |
| --- | --- | --- | --- |
|  | **BDOT10k** | **Spatial analysis** | **GHS-POP** |
| ***Supportive*** | | | |
| Low distance to city centre |  | ● |  |
| Big density population |  |  | ● |
| Places of stops of city’s public transport | ● |  |  |
| Close proximity to bus and railway stations | ● |  |  |
| Close proximity to bicycle routes, roads, parking lots | ● |  |  |
| Close proximity to important buildings (commercial and service buildings, cultural, religious, schools, museums, monuments) | ● | ● |  |
| ***Non-supportive*** | | | |
| Locations of railway | ● |  |  |
| Places of watercourses and reservoirs, swamps and wetlands | ● |  |  |
| Places of buildings (in the sense of their actual occurrence in the terrain) | ● |  |  |
| Places of cemeteries, military training grounds, industrial areas, landfills and workings | ● |  |  |
