# Supplementary material for: Optimizing bike-sharing station locations: A machine learning and artificial neural networks approach using geospatial and demographic data
Source: PLoS One. 2026 May 19;21(5):e0349339. doi: 10.1371/journal.pone.0349339 (PMC13186375; doi:10.1371/journal.pone.0349339)
Supplement: S2 Table — (DOCX) [file pone.0349339.s002.docx]

|  |  | ***actual*** | |
| --- | --- | --- | --- |
| ***predicted*** | ***Total: 10890*** | ***0*** | ***1*** |
|  | ***0*** | 6850 | 13 |
|  | ***1*** | 3906 | 121 |
