# Supplementary material for: Optimizing bike-sharing station locations: A machine learning and artificial neural networks approach using geospatial and demographic data
Source: PLoS One. 2026 May 19;21(5):e0349339. doi: 10.1371/journal.pone.0349339 (PMC13186375; doi:10.1371/journal.pone.0349339)
Supplement: S7 Table — (DOCX) [file pone.0349339.s007.docx]

| Method reference | Accuracy of model | Precision of model | Sensitivity of model | Specificity of model |
| --- | --- | --- | --- | --- |
| Case 1 | 0.6401 | 0.6369 | 0.9981 | 0.0300 |
| Case 2 | 0.9748 | 0.9860 | 0.9885 | 0.0679 |
| Case 3 | 0.6757 | 0.6740 | 0.9964 | 0.0299 |
| Case 4 | 0.9705 | 0.9793 | 0.9907 | 0.1357 |
| Case 5 | 0.9777 | 0.9876 | 0.9898 | 0.1529 |
