# Supplementary material for: Optimizing bike-sharing station locations: A machine learning and artificial neural networks approach using geospatial and demographic data
Source: PLoS One. 2026 May 19;21(5):e0349339. doi: 10.1371/journal.pone.0349339 (PMC13186375; doi:10.1371/journal.pone.0349339)
Supplement: S8 Table — (DOCX) [file pone.0349339.s008.docx]

| **Number** | **Original name** | **Meaning** | **Value of correlation** |
| --- | --- | --- | --- |
| 1 | *w_d_centr* | Distance from city centre | 0.49 |
| 2 | *w_au_tra* | Places of stops of city’s public transport | 0.18 |
| 3 | *w_drogi* | Proximity to roads | 0.11 |
| 4 | *w_gest1* | Population density | 0.06 |
| 5 | *w_szk* | Proximity to schools | 0.04 |
