# Supplementary material for: Optimizing bike-sharing station locations: A machine learning and artificial neural networks approach using geospatial and demographic data
Source: PLoS One. 2026 May 19;21(5):e0349339. doi: 10.1371/journal.pone.0349339 (PMC13186375; doi:10.1371/journal.pone.0349339)
Supplement: S9 Table — (DOCX) [file pone.0349339.s009.docx]

|  | **Warsaw** | **Lodz** |
| --- | --- | --- |
| **0-100 m** | 23% | 27% |
| **0-200 m** | 53% | 45% |
| **0-300 m** | 67% | 60% |
