# Supplementary material for: Optimizing bike-sharing station locations: A machine learning and artificial neural networks approach using geospatial and demographic data
Source: PLoS One. 2026 May 19;21(5):e0349339. doi: 10.1371/journal.pone.0349339 (PMC13186375; doi:10.1371/journal.pone.0349339)
Supplement: S10 Table — (DOCX) [file pone.0349339.s010.docx]

|  | **Warsaw** | | **Lodz** | | **Rzeszow** |
| --- | --- | --- | --- | --- | --- |
|  | **Actual** | **Predicted** | **Actual** | **Predicted** | **Predicted** |
| **GMI** | 0.007 | 0.122 | -0.002 | 0.093 | 0.572 |
| **p-value** | 0.021 | 0.000 | 0,695 | 0.000 | 0.000 |
| **z-score** | 2.319 | 40.871 | -0,392 | 16.009 | 94.402 |
