# Supplementary material for: Optimizing bike-sharing station locations: A machine learning and artificial neural networks approach using geospatial and demographic data
Source: PLoS One. 2026 May 19;21(5):e0349339. doi: 10.1371/journal.pone.0349339 (PMC13186375; doi:10.1371/journal.pone.0349339)
Supplement: S11 Table — (DOCX) [file pone.0349339.s011.docx]

|  | **Warsaw** | **Lodz** |
| --- | --- | --- |
| **Accuracy** | 0.906 | 0.864 |
| **AUC** | 0.651 | 0.340 |
| **MSE** | 0.084 | 0.121 |
