# Supplementary material for: Optimizing bike-sharing station locations: A machine learning and artificial neural networks approach using geospatial and demographic data
Source: PLoS One. 2026 May 19;21(5):e0349339. doi: 10.1371/journal.pone.0349339 (PMC13186375; doi:10.1371/journal.pone.0349339)
Supplement: S12 Table — (DOCX) [file pone.0349339.s012.docx]

|  | **Coverage distance = 300 m** | | **Coverage distance = 500 m** | | **Coverage distance = 700 m** | |
| --- | --- | --- | --- | --- | --- | --- |
|  | **Warsaw** | **Lodz** | **Warsaw** | **Lodz** | **Warsaw** | **Lodz** |
| **MAE** | 225.17 m | 262.54 m | 225.17 m | 262.54 m | 225.17 m | 262.54 m |
| **RMSE** | 284.86 m | 353.19 m | 284.86 m | 353.19 m | 284.86 m | 353.19 m |
| **Coverage** | 55.49% | 60.00% | 73.52% | 77.86% | 81.41% | 88.57% |
| $\boldsymbol{L}_{\boldsymbol{total}}$ | 0.182 | 0.195 | 0.128 | 0.141 | 0.104 | 0.109 |
