# Supplementary material for: Optimizing bike-sharing station locations: A machine learning and artificial neural networks approach using geospatial and demographic data
Source: PLoS One. 2026 May 19;21(5):e0349339. doi: 10.1371/journal.pone.0349339 (PMC13186375; doi:10.1371/journal.pone.0349339)
Supplement: S13 Table — (DOCX) [file pone.0349339.s013.docx]

|  | **Threshold = 1000 m** | | **Threshold = 2000 m** | | **Threshold = 3000 m** | |
| --- | --- | --- | --- | --- | --- | --- |
|  | **Warsaw** | **Lodz** | **Warsaw** | **Lodz** | **Warsaw** | **Lodz** |
| **MAE** | 225.17 m | 262.54 m | 251.65 m | 362.15 m | 317.76 m | 463.47 m |
| **RMSE** | 284.86 m | 353.19 m | 350.46 m | 537.78 m | 546.07 m | 746.48 m |
| **Coverage** | 81.41% | 88.57% | 81.41% | 88.57% | 81.41% | 88.57% |
| $\boldsymbol{L}_{\boldsymbol{total}}$ | 0.104 | 0.109 | 0.129 | 0.208 | 0.235 | 0.369 |
