# Supplementary material for: Optimizing bike-sharing station locations: A machine learning and artificial neural networks approach using geospatial and demographic data
Source: PLoS One. 2026 May 19;21(5):e0349339. doi: 10.1371/journal.pone.0349339 (PMC13186375; doi:10.1371/journal.pone.0349339)
Supplement: S14 Table — (DOCX) [file pone.0349339.s014.docx]

| **Method reference** | **Model complexity** | | | **Computational efficiency** | | | |
| --- | --- | --- | --- | --- | --- | --- | --- |
|  | *Number of nodes* | *Number of leaves* | *Number of features* | *Training time [s]* | *Inference time [s]* | *CPU usage* | *RAM usage* |
| Case 1 | 55 | 28 | 15 | 0.456 | 0.020 | 61.80% | 91.10% |
|  | *Intercept* | *Number of coefficients* | *Number of features* |  | | | |
| Case 2 | 0.402 | 15 | 15 | 0.145 | 0.002 | 47.50% | 91.70% |
|  | *Total params* | *Trainable params* | *Optimizer params* | (for 200 epochs) | | | |
| Case 3 | 868 | 433 | 435 | 87.199 | 0.466 | 16.46% | 85.41% |
| Case 4 | 1301 | 433 | 868 | 84.013 | 0.423 | 15.32% | 80.22% |
| Case 5 | 1301 | 433 | 868 | 82.277 | 0.235 | 13.76% | 81.98% |
