# Supplementary material for: Optimizing bike-sharing station locations: A machine learning and artificial neural networks approach using geospatial and demographic data
Source: PLoS One. 2026 May 19;21(5):e0349339. doi: 10.1371/journal.pone.0349339 (PMC13186375; doi:10.1371/journal.pone.0349339)
Supplement: Attachment — Submitted filename: Response to Editor.docx [file pone.0349339.s016.docx]

**For research article “Optimizing bike-sharing station locations: a machine learning and artificial neural networks approach using geospatial and demographic data”**

| Response to Editor Comments | | |
| --- | --- | --- |
| **1. Summary** |  |  |
| We would like to thank you for spending your precious time on processing the manuscript and for giving us constructive suggestions to deeply improve the quality of the paper. Please find the detailed responses below and the corresponding revisions in track changes in the re-submitted files*.* We hope that our answers are satisfactory and in line with your comments. All comments have been answered and either implemented in the work or explained why no action was taken. Thanks to them the substantive value of the article was improved and we hope that this article will be published. | | |
| **2. Point-by-point response to Comments and Suggestions for Authors** | | |
| **Comments 1:** *Please ensure that your manuscript meets PLOS ONE's style requirements, including those for file naming. The PLOS ONE style templates can be found at*  [*https://journals.plos.org/plosone/s/file?id=wjVg/PLOSOne_formatting_sample_main_body.pdf*](https://journals.plos.org/plosone/s/file?id=wjVg/PLOSOne_formatting_sample_main_body.pdf) *and*  [*https://journals.plos.org/plosone/s/file?id=ba62/PLOSOne_formatting_sample_title_authors_affiliations.pdf*](https://journals.plos.org/plosone/s/file?id=ba62/PLOSOne_formatting_sample_title_authors_affiliations.pdf) | | |
| **Response 1**: Checked. The manuscript meets PLOS ONE's style requirements with double-spacing implemented as it was stated in the requirements file. | | |
| **Comments 2:** *In your Methods section, please provide additional information regarding the permits you obtained for the work. Please ensure you have included the full name of the authority that approved the field site access and, if no permits were required, a brief statement explaining why.* | | |
| **Response 2:** Comment applied. An appropriate statement in section Materials and Methods was added. | | |

**Comments 3:** *Please note that PLOS One has specific guidelines on code sharing for submissions in which author-generated code underpins the findings in the manuscript. In these cases, we expect all author-generated code to be made available without restrictions upon publication of the work. Please review our guidelines at* [*https://journals.plos.org/plosone/s/materials-and-software-sharing#loc-sharing-code*](https://journals.plos.org/plosone/s/materials-and-software-sharing#loc-sharing-code) *and ensure that your code is shared in a way that follows best practice and facilitates reproducibility and reuse.*

**Response 3:** The data was published in a figshare repository alongside other work files, which can be found under the following DOI link: 10.6084/m9.figshare.31566433. An appropriate instructions to the users of the code were written in the README file attached to the code files.

**Comments 4:** *Thank you for stating the following financial disclosure: [A publication fee will be funded by Military University of Technology, Faculty of Civil Engineering and Geodesy]. Please state what role the funders took in the study. If the funders had no role, please state: ""The funders had no role in study design, data collection and analysis, decision to publish, or preparation of the manuscript.""*

*If this statement is not correct you must amend it as needed.*

*Please include this amended Role of Funder statement in your cover letter; we will change the online submission form on your behalf.*

**Response 4:** An appropriate statement was added in the section Acknowledgements in manuscript and also in cover letter.

**Comments 5:** *Thank you for uploading your study's underlying data set. Unfortunately, the repository you have noted in your Data Availability statement does not qualify as an acceptable data repository according to PLOS's standards.*

*At this time, please upload the minimal data set necessary to replicate your study's findings to a stable, public repository (such as figshare or Dryad) and provide us with the relevant URLs, DOIs, or accession numbers that may be used to access these data. For a list of recommended repositories and additional information on PLOS standards for data deposition, please see* [*https://journals.plos.org/plosone/s/recommended-repositories*](https://journals.plos.org/plosone/s/recommended-repositories)

**Response 5:** The data was published in a figshare repository, which can be found under the following DOI link: 10.6084/m9.figshare.31566433.

**Comments 6:** *When completing the data availability statement of the submission form, you indicated that you will make your data available on acceptance. We strongly recommend all authors decide on a data sharing plan before acceptance, as the process can be lengthy and hold up publication timelines. Please note that, though access restrictions are acceptable now, your entire data will need to be made freely accessible if your manuscript is accepted for publication. This policy applies to all data except where public deposition would breach compliance with the protocol approved by your research ethics board. If you are unable to adhere to our open data policy, please kindly revise your statement to explain your reasoning and we will seek the editor's input on an exemption. Please be assured that, once you have provided your new statement, the assessment of your exemption will not hold up the peer review process.*

**Response 6:** The data was published in a figshare repository, which can be found under the following DOI link: 10.6084/m9.figshare.31566433.

**Comments 7:** *We note that Figures 1,2,9,10,11,12 and 13 in your submission contain map images which may be copyrighted. All PLOS content is published under the Creative Commons Attribution License (CC BY 4.0), which means that the manuscript, images, and Supporting Information files will be freely available online, and any third party is permitted to access, download, copy, distribute, and use these materials in any way, even commercially, with proper attribution. For these reasons, we cannot publish previously copyrighted maps or satellite images created using proprietary data, such as Google software (Google Maps, Street View, and Earth). For more information, see our copyright guidelines:* [*http://journals.plos.org/plosone/s/licenses-and-copyright*](http://journals.plos.org/plosone/s/licenses-and-copyright)*.*

*We require you to either (1) present written permission from the copyright holder to publish these figures specifically under the CC BY 4.0 license, or (2) remove the figures from your submission:*

*1. You may seek permission from the original copyright holder of Figures 1,2,9,10,11,12 and 13 to publish the content specifically under the CC BY 4.0 license.*

*We recommend that you contact the original copyright holder with the Content Permission Form (*[*http://journals.plos.org/plosone/s/file?id=7c09/content-permission-form.pdf*](http://journals.plos.org/plosone/s/file?id=7c09/content-permission-form.pdf)*) and the following text:*

*“I request permission for the open-access journal PLOS ONE to publish XXX under the Creative Commons Attribution License (CCAL) CC BY 4.0 (*[*http://creativecommons.org/licenses/by/4.0/*](http://creativecommons.org/licenses/by/4.0/)*). Please be aware that this license allows unrestricted use and distribution, even commercially, by third parties. Please reply and provide explicit written permission to publish XXX under a CC BY license and complete the attached form.”*

*Please upload the completed Content Permission Form or other proof of granted permissions as an ""Other"" file with your submission.*

*In the figure caption of the copyrighted figure, please include the following text: “Reprinted from [ref] under a CC BY license, with permission from [name of publisher], original copyright [original copyright year].”*

*2. If you are unable to obtain permission from the original copyright holder to publish these figures under the CC BY 4.0 license or if the copyright holder’s requirements are incompatible with the CC BY 4.0 license, please either i) remove the figure or ii) supply a replacement figure that complies with the CC BY 4.0 license. Please check copyright information on all replacement figures and update the figure caption with source information. If applicable, please specify in the figure caption text when a figure is similar but not identical to the original image and is therefore for illustrative purposes only.*

*The following resources for replacing copyrighted map figures may be helpful:*

*USGS National Map Viewer (public domain):* [*http://viewer.nationalmap.gov/viewer/*](http://viewer.nationalmap.gov/viewer/)

*The Gateway to Astronaut Photography of Earth (public domain):* [*http://eol.jsc.nasa.gov/sseop/clickmap/*](http://eol.jsc.nasa.gov/sseop/clickmap/)

*Maps at the CIA (public domain):* [*https://www.cia.gov/library/publications/the-world-factbook/index.html*](https://www.cia.gov/library/publications/the-world-factbook/index.html) *and* [*https://www.cia.gov/library/publications/cia-maps-publications/index.html*](https://www.cia.gov/library/publications/cia-maps-publications/index.html)

*NASA Earth Observatory (public domain):* [*http://earthobservatory.nasa.gov/*](http://earthobservatory.nasa.gov/)

*Landsat:* [*http://landsat.visibleearth.nasa.gov/*](http://landsat.visibleearth.nasa.gov/)

*USGS EROS (Earth Resources Observatory and Science (EROS) Center) (public domain):* [*http://eros.usgs.gov/#*](http://eros.usgs.gov/)

*Natural Earth (public domain):* [*http://www.naturalearthdata.com/*](http://www.naturalearthdata.com/)

**Response 7:** Base map used in Fig 1 was changed into the map obtained from the site recommended by you (https://www.usgs.gov/tools/national-map-viewer) as an appropriate one (with CC BY 4.0 licence). Base maps used in Fig 2a, Fig 11 and Fig 12 are obtained from Polish National Geoportal (<https://www.geoportal.gov.pl/pl/dane/panstwowy-rejestr-granic-prg/>) shared under the CC BY-SA 4.0 licence (<https://www.gov.pl/web/archiwum-inwestycje-rozwoj/geoportal-informacja-przestrzenna-dla-kazdego>). Nonetheless, an appropriate permission to publish aforementioned figures under CC BY 4.0 licence was obtained from the owner of the data (Head Office of Geodesy and Cartography) and the permission document was attached to the submission. Base maps used in Fig 2b is obtained from Global Human Settlement Layer (https://human-settlement.emergency.copernicus.eu/index_op.php) and has been shared under the CC BY 4.0 licence (https://human-settlement.emergency.copernicus.eu/GHSLhowToCite.php). Other basemaps used in mentioned figures are created with the use of OpesStreetMap data (https://www.openstreetmap.org/), which uses Open Database Licence (<http://www.opendatacommons.org/licenses/odbl>). According to previously published via PLOS ONE articles, such as <https://doi.org/10.1371/journal.pone.0139970>; <https://doi.org/10.1371/journal.pone.0334627> and the newest <https://doi.org/10.1371/journal.pone.0343242>, it is possible to use OSM data in PLOS ONE manuscripts. To make this clear for article readers, an appropriate sentences about the sources of basemaps were added to descriptions of aforementioned figures in the manuscript. Therefore, we claim that no further action connected with licencing matters is required.

**Comments 8:** *Please include captions for your Supporting Information files at the end of your manuscript, and update any in-text citations to match accordingly. Please see our Supporting Information guidelines for more information:* [*http://journals.plos.org/plosone/s/supporting-information*](http://journals.plos.org/plosone/s/supporting-information)

**Response 8:** The comment was implemented in the manuscript as the Supporting Information section was added at the bottom of the manuscript and fulfilled.

**Comments 9:** *We notice that your supplementary file tables are included in the manuscript file. Please remove them and upload them with the file type 'Supporting Information'. Please ensure that each Supporting Information file has a legend listed in the manuscript after the references list.*

**Response 9:** The comment was implemented in the manuscript as tables were removed and made as supporting information. Nevertheless, please, note that this comment denies the instructions performed in your file:

<https://journals.plos.org/plosone/s/file?id=wjVg/PLOSOne_formatting_sample_main_body.pdf>

**Comments 10:** *If the reviewer comments include a recommendation to cite specific previously published works, please review and evaluate these publications to determine whether they are relevant and should be cited. There is no requirement to cite these works unless the editor has indicated otherwise. [Note: HTML markup is below. Please do not edit.]Reviewers' comments:Reviewer's Responses to Questions.*

**Response 10:** The reviewers comments were implemented in the work and answeared in a response letter. All needed changes have been made.
